# Associations of early pregnancy high-sensitivity C-reactive protein levels with subsequent gestational diabetes: A Finnish gestational diabetes study

**DOI:** 10.1186/s13098-025-01843-0

**Published:** 2025-07-16

**Authors:** Shilpa Lingaiah, Marja Vääräsmäki, Hanna Öhman, Eero Kajantie, Elina Keikkala, Sanna Mustaniemi

**Affiliations:** 1 Research Unit of Clinical Medicine, Medical Research Centre Oulu, Oulu University Hospital and University of Oulu, P. O. Box 5000, Oulu, FI-90014 Finland; 2 Department of Public Health, Welfare Epidemiology and Monitoring Unit, Finnish Institute for Health and Welfare, Helsinki/Oulu, Finland; 3 Biobank Borealis of Northern Finland, Oulu University Hospital, Oulu, Finland; 4 Faculty of Medicine, University of Oulu, Oulu, Finland; 5 Children’s Hospital, University of Helsinki and Helsinki University Hospital, Helsinki, Finland; 6 Department of Clinical and Molecular Medicine, Norwegian University of Science and Technology, Trondheim, Norway

**Keywords:** Gestational diabetes, Oral glucose tolerance test, High-sensitivity C-reactive protein, Inflammation

## Abstract

**Background:**

High sensitivity C-reactive protein (hsCRP) is a sensitive marker of subclinical inflammation and has been proposed as a marker for predicting gestational diabetes in early pregnancy. However, data concerning the levels of hsCRP in early pregnancy are inconsistent. We aimed to determine hsCRP levels in early pregnancy and to examine the associations between hsCRP levels and the number of abnormal values in the oral glucose tolerance test and the type of hyperglycemia.

**Methods:**

Early pregnancy serum hsCRP levels were analyzed in 776 women with gestational diabetes and 856 pregnant controls without diabetes. The mean gestational age at sampling was 10.7 weeks.

**Results:**

Early pregnancy hsCRP levels were higher in women who later developed gestational diabetes compared to controls (geometric mean 3.71 mg/L vs. 2.62 mg/L). After adjustments, including those for maternal age and pre-pregnancy body mass index, the results remained significant. When compared to the lowest quartile of hsCRP, women in the other quartiles had 1.5-fold odds for gestational diabetes after adjustments. Furthermore, hsCRP levels were higher in women with two or three abnormal oral glucose tolerance test values compared to those with one abnormal value and among women with postprandial hyperglycemia compared to those with only fasting hyperglycemia.

**Conclusion:**

Early pregnancy serum hsCRP levels were associated with subsequent gestational diabetes, and most with a higher number of abnormal values and postprandial hyperglycemia in the oral glucose tolerance test. These findings highlight the role of chronic inflammation and clustering of metabolic risk factors involved in gestational diabetes and its severity.

**Supplementary Information:**

The online version contains supplementary material available at 10.1186/s13098-025-01843-0.

## Background

Gestational diabetes (GDM), defined as glucose intolerance of variable degree resulting in hyperglycemia with onset or first recognition during pregnancy, is a common pregnancy complication with a prevalence of 7 − 28% worldwide [1]. Apart from the short-term maternal and perinatal outcomes, GDM is also associated with long-term adverse health outcomes for both the mother and the child [2–5]. Women with GDM have a ten-fold higher risk of developing type 2 diabetes (T2D) compared to women with no GDM, and one-third of women develop T2D within 15 years after a GDM pregnancy [6, 7]. GDM also predisposes women to an increased risk of metabolic syndrome, incident hypertension and cardiovascular diseases (CVD) [8–10]. Of note, GDM increases the risk of CVD independent of intercurrent T2D [11]. The most commonly used method to diagnose GDM is a two-hour 75 g oral glucose tolerance test (OGTT), and the diagnosis is based on one or more abnormal values in the OGTT. Further, a higher number of abnormal values in the OGTT has been associated with increased risks of adverse maternal and neonatal outcomes [12–14]. Thus, the number of abnormal values in the OGTT can reflect the severity of GDM.

Normal pregnancy is characterized by physiological insulin resistance with a compensatory increase in insulin secretion [15]. Women who develop GDM often have chronic insulin resistance prior to conception, which is further exacerbated during pregnancy, and hyperglycemia develops when pancreatic compensatory mechanisms are insufficient [16, 17]. Besides hyperglycemia, insulin resistance is also linked to inflammation [18]. It has been suggested that inflammatory dysregulation may manifest as early as the first trimester in women who later develop GDM [19].

Chronic inflammation is known to play an important role in the development of metabolic diseases [20–22]. High sensitivity C-reactive protein (hsCRP) is a sensitive marker of subclinical inflammation, and elevated hsCRP levels are associated with prediabetes, T2D and CVD [23–25]. hsCRP has also been proposed as a marker for predicting GDM in early pregnancy [26, 27]. However, data concerning the levels of C-reactive protein in early pregnancy are inconsistent, with studies reporting higher [26–28] as well as unchanged levels [29, 30] among women who eventually develop GDM. There is also a paucity of studies examining the association of hsCRP with the severity of GDM. This study aims to determine the associations of early pregnancy hsCRP with subsequent GDM. Additionally, we aimed to study the associations between hsCRP levels and the severity of GDM, defined by the number of abnormal values in the oral glucose tolerance test (OGTT) and the type of hyperglycemia.

## Methods

### Study population

This case-control study is based on the clinical-genetic arm of the Finnish Gestational Diabetes (FinnGeDi) study as described previously [31]. Briefly, 1146 women with GDM and 1066 non-diabetic controls were selected from seven Finnish delivery hospitals between 1 February 2009 and 31 December 2012. Women with GDM were recruited at the hospitals before delivery, and the next consenting non-diabetic mother giving birth in the same hospital was chosen as a control. Women with pre-pregnancy diabetes or multiple pregnancies were excluded from the study. Informed signed consent was obtained from all the participants. The Ethics Committee of the Northern Ostrobothnia Hospital District approved the study (Reference number 33/2008).

Based on the Finnish national Current Care Guidelines [32], a comprehensive screening for GDM is performed in all women at 24th–28th weeks of gestation, except for those at very low risk for GDM by a two-hour 75 g OGTT. The low-risk group comprises primiparous women under 25 years of age with body mass index (BMI) < 25 kg/m^2^ without a family history of T2D and multiparous women under 40 years with BMI < 25 kg/m^2^ without previous GDM and macrosomic births [32, 33]. In high-risk women (previous GDM, pre-pregnancy BMI > 35 kg/m^2^, glucosuria in early pregnancy, T2D in a first-degree relative, systemic glucocorticoid treatment, or polycystic ovary syndrome), OGTT is performed at 12th–16th weeks of gestation and, if normal, repeated at 24th–28th weeks. The OGTT is performed after a 12-hour overnight fast in the laboratory nearest to the woman’s residence. Blood samples are drawn from the antecubital vein into fluoride citrate tubes and analyzed within 24 h in a local laboratory using commercial enzymatic assays. The involved laboratories in the study were accredited laboratories under the ISO15189:2012 standard and had quality management systems. The laboratories performed regular internal quality control checks with controls of known concentrations and were also involved in external quality control schemes. The cut-off values for the glucose concentrations were set according to the Finnish guidelines as follows: fasting ≥ 5.3 mmol/L, one hour ≥ 10.0 mmol/L and two hours ≥ 8.6 mmol/L. GDM was diagnosed when one or more OGTT values were abnormal. Women with one or several abnormal OGTT values received individualized dietary and lifestyle counselling in maternity clinics and began glucose self-monitoring thereafter. If self-monitored plasma glucose concentrations repeatedly exceeded the target levels (i.e., < 5.5 mmol/L fasting and < 7.8 mmol/L one hour postprandial) after dietary and lifestyle interventions, pharmacological therapy was considered.

### Clinical and register data

The study participants completed background questionnaires, including information on their lifestyle habits, and medical and family histories. Pregnancy and delivery data were collected from the hospital and maternal welfare clinic records, which were combined with individually linked register data obtained from the Finnish medical birth register.

Information on maternal age at delivery and parity was obtained from the Finnish medical birth register. Smoking during pregnancy was obtained from the questionnaire and the Finnish medical birth register. Pre-pregnancy BMI was calculated from self-reported maternal height and pre-pregnancy weight obtained from the maternal welfare clinic records. Gestational weight gain was calculated as the difference between the pre-pregnancy weight and the weight at the last antenatal visit after 35 weeks of gestation. Based on the questionnaire data, educational attainment was classified as basic or less (≤ 9 years), upper secondary (10–12 years), lower-level tertiary (13–15 years) and upper-level tertiary (> 15 years).

Data on blood pressure were obtained from the hospital and maternal welfare clinic records. Chronic hypertension was defined as a systolic blood pressure ≥ 140 mmHg or diastolic blood pressure ≥ 90 mmHg or the use of antihypertensive medication before 20 weeks of gestation, while gestational hypertension was defined as blood pressure elevation after 20 weeks of gestation. Pre-eclampsia was specified as elevated blood pressure after 20 weeks of gestation with proteinuria (≥ 300 mg/day or two ≥ 1 + readings on a dipstick test) or as chronic hypertension with proteinuria. Large-for-gestational age was defined as birth weight over the 90th percentile for sex and gestational age.

Since autoimmune and infectious diseases are associated with increased levels of hsCRP, the number of women with these diagnoses was determined in our study population. Autoimmune and/or infectious diseases diagnoses were ascertained based on the diagnosis codes obtained from the comprehensive and individual-level healthcare data repositories of the Finnish Institute for Health and Welfare: *(i)* the Care Register for Social Welfare and Health Care (Hilmo), which includes both inpatient data (hospitalizations and procedures/interventions with codes) and hospital outpatient contacts (scheduled and emergency care specialist visits) and *(ii)* the Register of Primary Health Care Visits (Avohilmo) which includes all primary healthcare contacts at the healthcare centers [34, 35]. Both registers include information on the date of contact (visit or admission) and all diagnoses made, classified according to International Statistical Classification of Diseases and Related Health Problems, Tenth Revision (ICD-10).

### Serum samples and laboratory analysis

The early pregnancy serum samples were obtained from the Finnish Maternity Cohort, a nationwide collected cohort of leftover serum samples from routine infectious disease screening performed during early pregnancy. Hence, the sampling timing was not standardized and fasting before sampling was not required. All samples were stored at -25 °C in the Biobank Borealis of Northern Finland. Of the 2212 women included in the FinnGeDi case-control cohort, 192 did not have serum sample available for analysis (no sample in the biobank, *n* = 124, and depleted sample, *n* = 68; total *n* = 192). The characteristics of participants with missing samples did not differ significantly from those included in the analysis with available serum samples. Samples drawn after 20 weeks of gestation (*n* = 22) were excluded. The mean gestational age at sampling was 10.7 weeks (standard deviation [SD] 2.1).

**Fig. 1:**
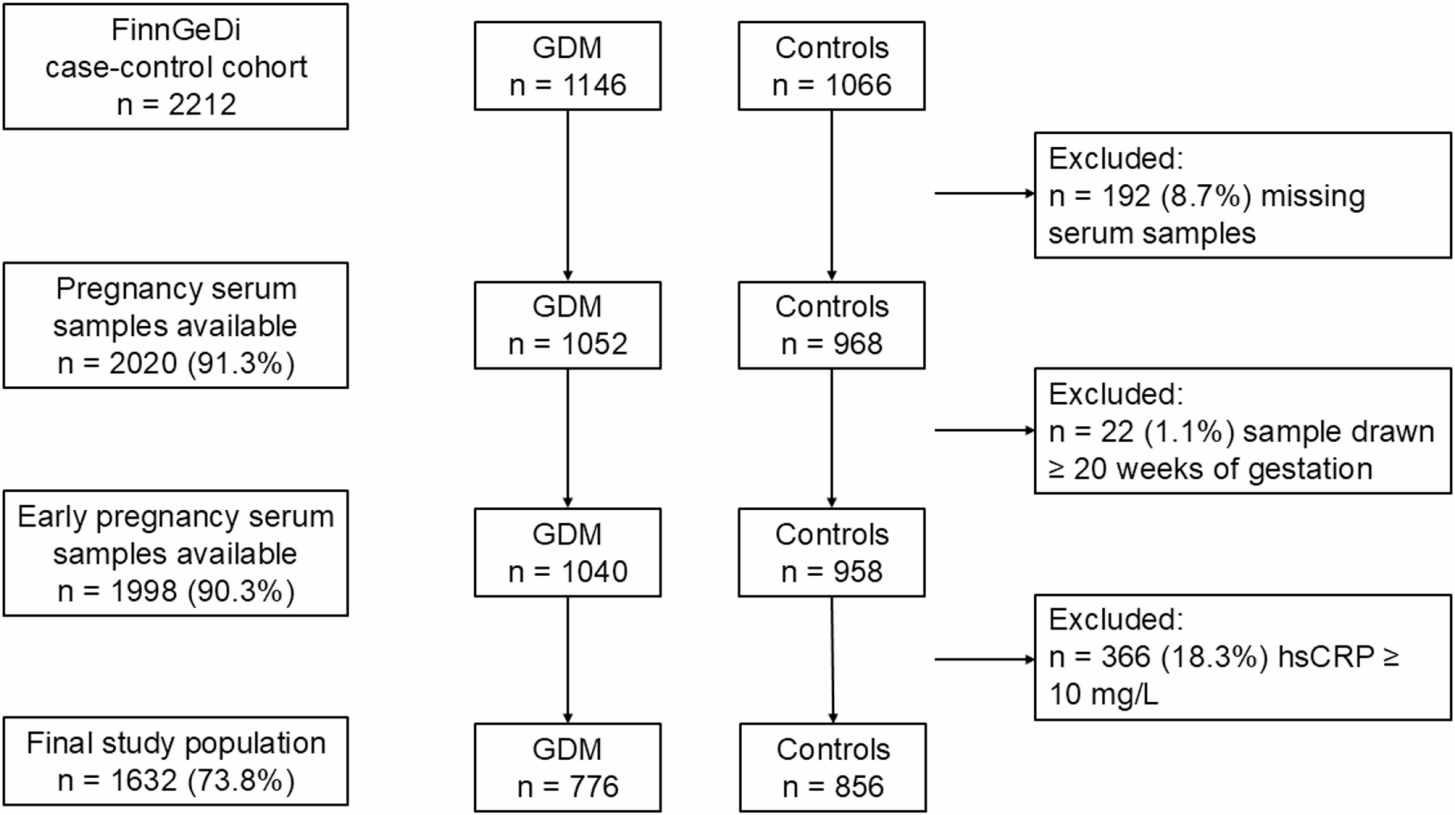
Flowchart of the study population. FinnGeDi, Finnish Gestational Diabetes study; GDM, gestational diabetes, hsCRP, high-sensitivity C-reactive protein

The samples were analyzed for hsCRP using hsCRP ELISA kits, according to the manufacturer’s instructions (IBL International GmbH, Hamburg, Germany), by Biobank Borealis of Northern Finland, Oulu, Finland. Personnel carrying out laboratory analyses were blinded to the GDM status and other phenotypic data of the participants. Participants with a hsCRP ≥ 10 mg/L (*n* = 336) were excluded from the present study. The cut-off level of ≥ 10 mg/L was chosen as hsCRP levels over this are likely to indicate active infection or acute inflammation, rather than low-grade chronic inflammation [36–38]. The final study population consisted of 1632 participants (776 with GDM and 856 controls) (Fig. 1).

Autoimmune disease diagnosis was made irrespective of the sampling time, whereas the infectious disease diagnosis was made if it occurred within one month before the sample collection, including the sampling date. There were 127 participants (68 among women with GDM and 59 among control women) with either autoimmune disease or infectious disease within one month before sampling. The ICD-10 codes for the diagnosis of autoimmune and infectious diseases are detailed in Supplementary Tables 1 and 2.

### Statistical methods

Statistical analyses were performed using SPSS 29.0. Continuous variables were reported as means and SDs, and categorical variables as frequencies and percentages. The differences in the baseline characteristics were analyzed using the Student’s *t*-test for continuous variables and the χ^2^ test for categorical variables. The values of hsCRP were logarithmically transformed to normalize distribution, and the distribution described in terms of geometric means and geometric SDs. Linear regression models (mean differences with 95% confidence intervals [CIs]) were used to compare hsCRP levels between women with GDM and controls.

The levels of hsCRP were stratified by quartiles. To determine the association of hsCRP with GDM, odds ratios (ORs) with 95% CI per quartile (Q2–Q4) were calculated using the lowest quartile (Q1) as a reference. Model 1 was adjusted for gestational weeks at sampling and delivery hospital. Model 2 was adjusted for Model 1 together with maternal age and educational attainment. Model 3 was adjusted for Model 2 and pre-pregnancy BMI. Categorical variables were added as dummy-coded, with a separate dummy variable indicating missing values. Further, the associations between hsCRP levels and the number of abnormal values in the OGTT (one vs. several), type of hyperglycemia (fasting, postprandial or both) and the treatment of GDM (diet or pharmacologically treated) were analyzed and only the participants with GDM were included the linear regression. When the type of hyperglycemia was analyzed, continuous OGTT values were used. Sensitivity analyses were performed after excluding those with autoimmune or infectious disease (*n* = 127).

The directed acyclic graph summarizing the causality between hsCRP and GDM, and potential confounding variables used in the regression analyses is shown in Supplementary Fig. 1. The study was sufficiently powered to identify small differences between the study groups. With a power of 0.80 and a significance level of 0.05, we were able to detect a difference of 0.13 SD in hsCRP between women with GDM and the controls.

## Results

### Characteristics of the study population

The clinical characteristics of the study participants are shown in Table 1. Women with GDM were older and had higher pre-pregnancy BMIs compared to the controls. Educational attainment and smoking during pregnancy did not differ between women with GDM and controls. Women with GDM had more often hypertensive disorders, including chronic and gestational hypertension as well as pre-eclampsia. Of the women with GDM, 136 (17.9%) were medically treated, including 125 (16.5%) women treated with insulin and 17 (2.3%) with metformin. Autoimmune or infectious disease diagnoses was comparable between women with GDM and controls.

**Table 1:** Baseline characteristics of the study participants

Baseline characteristics	GDM (*n* = 776)	Controls (*n* = 856)	No. of missing (GDM/Controls)	*p* value
Age at delivery, years	32.1 (5.4)	29.6 (5.1)	-	< 0.001
Primiparity	438 (56.4%)	443 (51.8%)	-	0.058
Pre-pregnancy weight, kg	72.9 (15.3)	63.3 (10.9)	1/0	< 0.001
Height, cm	165.4 (5.8)	165.0 (6.0)	-	0.231
Pre-pregnancy BMI, kg/m^2^	26.7 (5.2)	23.1 (3.6)	1/0	< 0.001
Gestational weight gain, kg	12.9 (5.5)	14.7 (4.9)	63/25	< 0.001
Educational attainment			70/106	0.080
Basic or less	46 (6.5%)	35 (4.7%)		
Upper secondary	314 (44.5%)	330 (44.0%)		
Lower-level tertiary	183 (25.9%)	190 (25.3%)		
Upper-level tertiary	163 (23.0%)	195 (26.0%)		
Smoking during pregnancy	115 (14.8%)	127 (14.9%)	1/1	0.993
Chronic hypertension	120 (15.5%)	35 (4.1%)	1/0	< 0.001
Gestational hypertension	151 (19.5%)	122 (14.3%)	1/0	< 0.001
Pre-eclampsia	39 (5.0%)	20 (2.3%)	1/0	< 0.001
Number of abnormal values in OGTT			31	
One	451 (60.5%)			
Two	226 (30.3%)			
Three	68 (9.1%)			
Antidiabetic medication				
Insulin	125 (16.5%)		17	
Metformin	17 (2.3%)		24	
Any autoimmune or infectious disease diagnosis	68 (8.8%)	59 (6.9%)	-	0.159

Data shown as mean (standard deviation) or number (percentage). *p* values based on the student’s *t*-test or χ ^2^ test

GDM, gestational diabetes; OGTT, oral glucose tolerance test

### Associations of hsCRP with GDM

Early pregnancy hsCRP levels were higher in women who developed GDM compared to women with no GDM (geometric mean 3.71 mg/L vs. 2.62 mg/L) (Table 2). After adjustments for covariates in Model 1 (gestational weeks at sampling and delivery hospital) and Model 2 (Model 1 + maternal age and educational attainment), hsCRP levels were 27.9% and 25.0% higher, respectively, in the GDM group than in controls. After further adjustments for pre-pregnancy BMI (Model 3), the mean difference was still significant but substantially attenuated, being 8.1%.

**Table 2:** Differences in hsCRP levels in women with GDM and non-diabetic controls

	GDM	Controls		
	Geometric mean (SD)^1^	Geometric mean (SD)^1^	Mean difference (95% CI)	*p* value
hsCRP, mg/L	3.71 (2.00%)	2.62 (2.10%)		
Model 1			27.9% (22.3 to 33.5%)	< 0.001
Model 2			25.0% (19.3 to 30.8%)	< 0.001
Model 3			8.1% (2.5 to 13.8%)	0.005

^1^The geometric mean is the n^th^ root of the product of n values. Geometric SDs correspond to the percent increase in the variable corresponding to a change of one SD unit in the logarithm of the variable

Model 1: Linear regression adjusted for gestational weeks at sampling and delivery hospital

Model 2: Linear regression adjusted for Model 1 + maternal age and educational attainment

Model 3: Linear regression adjusted for Model 2 + pre-pregnancy BMI

**Fig. 2:**
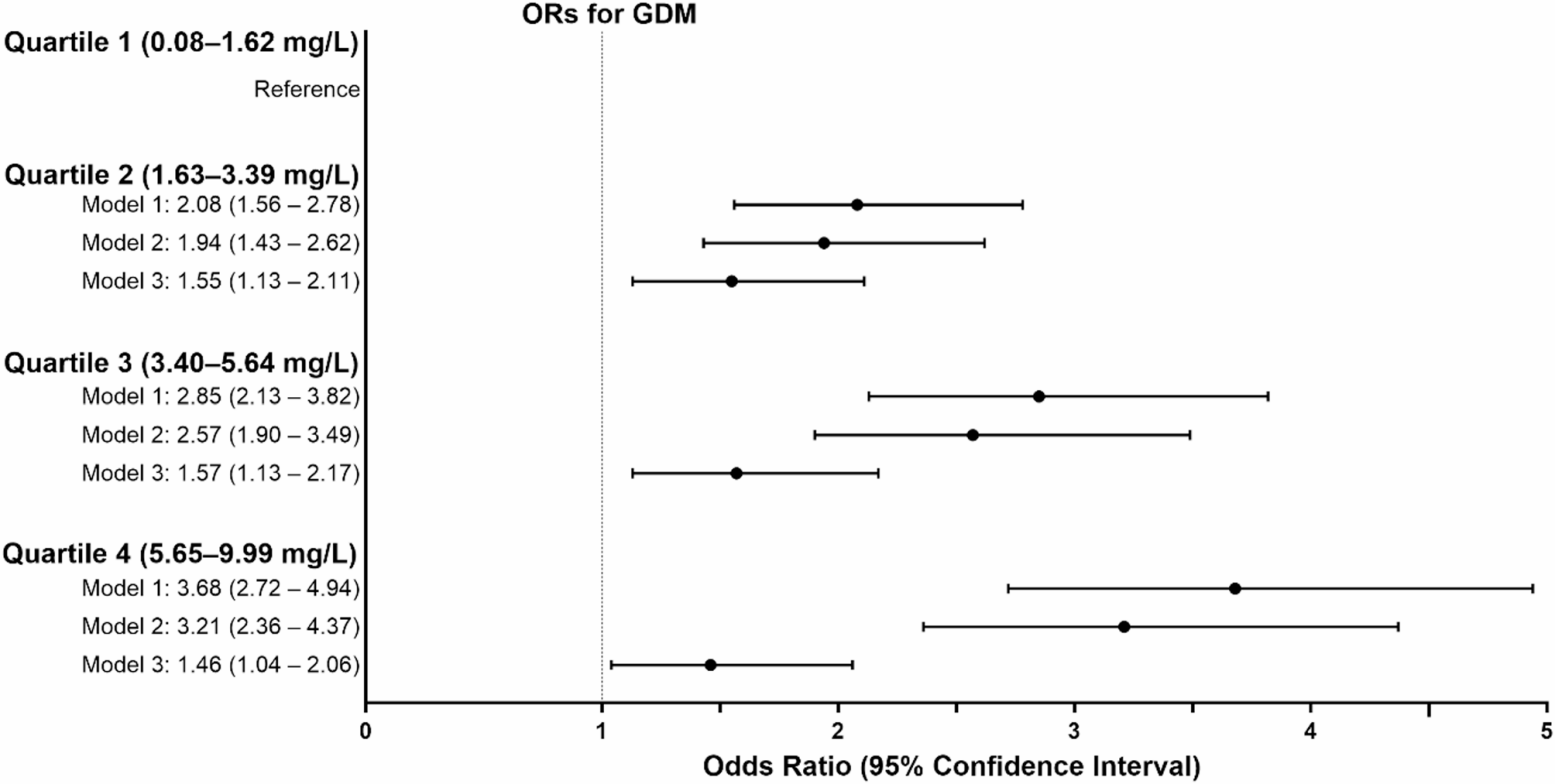
Odds ratios (ORs) and 95% confidence intervals per quartile of high sensitivity C-reactive protein for gestational diabetes (GDM). Model 1: Adjusted for gestational weeks at sampling and delivery hospital; Model 2: Model 1 + maternal age and educational attainment; Model 3: Model 2 + pre-pregnancy BMI

When ORs per hsCRP quartiles were assessed for GDM, and the lowest quartile was used as the reference, in Model 1, hsCRP showed 2.08-, 2.85- and 3.68-fold odds for GDM in quartiles 2, 3 and 4, respectively. The results remained significant after further adjustments for pre-pregnancy BMI (Model 3), but the odds for GDM were slightly attenuated (Fig. 2). Further, sensitivity analyses after excluding women with any autoimmune or infectious diseases did not change the results.

### Associations of hsCRP with the number of abnormal values in OGTT and the type of hyperglycemia

The levels of hsCRP were higher in women with two or three abnormal values in the OGTT compared to those with only one abnormal value (Table 3). Further, after adjustments for covariates in Model 1 and Model 2, hsCRP levels were still 18.0% and 17.2% higher, respectively. After adjustments for pre-pregnancy BMI in Model 3, the mean difference was decreased to 9.7%.

**Table 3:** Differences in hsCRP among women with GDM with one or two/three abnormal values in OGTT

	One abnormal value (*n* = 451)	Two/three abnormal values (*n* = 294)		
	Geometric mean (SD)^1^	Geometric mean (SD)^1^	Mean difference (95% CI)	*p* value
hsCRP, mg/L	3.44 (2.73%)	4.16 (2.99%)		
Model 1			18.0% (9.9 to 26.0%)	< 0.001
Model 2			17.2% (19.1 to 25.4%)	< 0.001
Model 3			9.7% (2.1 to 17.2%)	0.012

^1^The geometric mean is the n^th^ root of the product of n values. Geometric SDs correspond to the percent increase in the variable corresponding to a change of one SD unit in the logarithm of the variable

Model 1: Linear regression adjusted for gestational weeks at sampling and delivery hospital

Model 2: Linear regression adjusted for Model 1 + maternal age and educational attainment

Model 3: Linear regression adjusted for Model 2 + pre-pregnancy BMI

When the levels of hsCRP were analyzed based on the type of hyperglycemia, the highest levels of hsCRP were observed among women with both fasting and postprandial hyperglycemia and the lowest among those with only fasting hyperglycemia (Table 4). Higher hsCRP levels were independently associated with higher postprandial glucose concentrations but not with fasting glucose concentrations (Table 5). There were no significant differences in the levels of hsCRP between women with diet versus pharmacologically treated GDM. The results remained unchanged when chronic hypertension was used as a covariate in the regression models (data not shown). hsCRP was not independently associated with higher risk for large-for-gestational age infants. The results were similar after excluding those with autoimmune or infectious diseases.

**Table 4:** Differences in hsCRP among women with GDM with fasting and postprandial hyperglycemia in OGTT

	Fasting hyperglycemia (*n* = 234)	Postprandial hyperglycemia (*n* = 309)	Both fasting and postprandial hyperglycemia (*n* = 205)
	Geometric mean (SD)^1^	Geometric mean (SD)^1^	Geometric mean (SD)^1^
hsCRP, mg/L	3.41 (3.97%)	3.54 (3.14%)	4.39 (3.48%)
			Mean difference (95% CI)
Both fasting and postprandial hyperglycemia vs. postprandial hyperglycemia			
Model 1	-	reference	19.8% (10.6 to 28.9%)
Model 2	-	reference	19.3% (9.9 to 28.6%)
Model 3	-	reference	4.3% (-4.7–13.4%)
Both fasting and postprandial hyperglycemia vs. fasting hyperglycemia			
Model 1	reference	-	21.5% (11.3 to 31.7%)
Model 2	reference	-	20.5% (10.1 to 30.9%)
Model 3	reference	-	14.8% (5.4 to 24.2%)
Postprandial hyperglycemia vs. fasting hyperglycemia			
Model 1	reference	2.7% (-6.8–12.2%)	-
Model 2	reference	3.4% (-6.1 to 12.9%)	-
Model 3	reference	14.9% (6.0 to 23.8%)	-

^1^The geometric mean is the n^th^ root of the product of n values. Geometric SDs correspond to the percent increase in the variable corresponding to a change of one SD unit in the logarithm of the variable

Model 1: Linear regression adjusted for gestational weeks at sampling and delivery hospital

Model 2: Linear regression adjusted for Model 1 + maternal age and educational attainment

Model 3: Linear regression adjusted for Model 2 + pre-pregnancy BMI

**Table 5:** hsCRP levels and its association with glucose values in the OGTT among women with GDM

	Mean difference (95% CI)	*p* value
	hsCRP, mg/L	
Fasting glucose, mmol/L		
Model 1	11.3% (3.7–18.6%)	0.003
Model 2	10.5% (3.1–17.8%)	0.005
Model 3	-3.4% (-11.1–4.4%)	0.398
1-hour glucose, mmol/L		
Model 1	45.2% (20.9–69.6%)	< 0.001
Model 2	43.5% (19.2–67.8%)	< 0.001
Model 3	52.3% (25.4–79.2%)	< 0.001
2-hours glucose, mmol/L		
Model 1	52.1% (30.5–73.6%)	< 0.001
Model 2	50.4% (28.8–71.9%)	< 0.001
Model 3	55.1% (31.3–7.9%)	< 0.001

Model 1: Linear regression adjusted for gestational weeks at sampling and delivery hospital

Model 2: Linear regression adjusted for Model 1 + maternal age and educational attainment

Model 3: Linear regression adjusted for Model 2 + pre-pregnancy BMI

## Discussion

In this case-control study, we found that serum hsCRP levels in early pregnancy are associated with subsequent GDM. As a novel finding, higher hsCRP levels were independently associated with the number of abnormal values and with postprandial hyperglycemia but not with fasting hyperglycemia in the OGTT.

Elevated hsCRP levels are indicative of chronic metabolic inflammation and maybe associated with CVD and T2D. Inflammation is known to play a crucial role in the development of T2D, and previous studies have shown that multiple inflammation markers, including hsCRP, are correlated with hyperglycemia [20, 39, 40]. Further, it has been shown that CRP levels are significantly increased in subjects with impaired glucose tolerance (IGT), and there is a strong association between increased hsCRP levels and IGT, implying that chronic inflammation is a risk factor for developing T2D [41–43].

Early pregnancy hsCRP has been proposed as a marker for predicting GDM. However, results regarding the levels of hsCRP in early pregnancy are conflicting, with studies reporting both increased and unchanged levels in women with GDM compared to controls [26–30]. This could be due to variations in the populations studied, varying GDM diagnostic criteria applied, study design, small sample sizes, and non-adjustment for various confounders. We found that serum hsCRP levels in early pregnancy were elevated in women with GDM compared to controls. The results were significantly attenuated after adjustment for pre-pregnancy BMI, likely because obesity and hsCRP are positively correlated [44, 45]. However, the difference remained significant even after adjusting for pre-pregnancy BMI and various other potential confounders. This suggests that chronic inflammation, with high hsCRP as its signal, may play a role in the development of GDM.

Few studies have examined the association of hsCRP levels with hyperglycemia among pregnant women. It has been reported that fasting plasma glucose and hsCRP in early pregnancy are correlated with the later development of GDM [27]. A positive correlation of hsCRP with fasting plasma glucose in the second trimester, which is further exaggerated in the third trimester, has also been reported [30]. The association between hsCRP and hyperglycemia has been studied earlier in prediabetes and newly diagnosed/onset T2D. Elevated levels of hsCRP have been reported in subjects with IGT, and a significant association between hsCRP levels and fasting plasma glucose and two-hour postprandial glucose has been found [41, 46]. Also, increased levels of hsCRP have been reported in subjects with impaired fasting glucose and IGT, independent of BMI [47]. On the contrary, it has also been reported that no significant difference was found in mean hsCRP level between impaired fasting glucose and IGT [48].

We found that the concentrations of hsCRP were higher in women with several abnormal values in the OGTT compared to those with only one abnormal value. Further, hsCRP levels were higher among women with postprandial hyperglycemia compared to fasting hyperglycemia. These findings have not been reported previously. Higher hsCRP levels were associated with higher postprandial glucose concentrations, independent of obesity. This is in line with the results of a previous study among non-pregnant population [47]. Even though hsCRP levels were associated with a higher number of abnormal values and postprandial hyperglycemia in the OGTT, it was not associated with higher odds for the need for pharmacological therapy or large-for-gestational age. Over 80% of women with GDM in our study achieved their glucose targets with diet al.one. Thus, the relatively small number of participants receiving pharmacological therapy may have limited the statistical power to detect a significant association between hsCRP levels and these outcomes.

Several plausible pathophysiological mechanisms may explain the differential association between hsCRP and the type of hyperglycemia. Postprandial hyperglycemia, more than continuous hyperglycemia, triggers a transient increase in circulating proinflammatory cytokines, including tumor necrosis factor-α (TNF-α) and interleukin-6 (IL-6) through oxidative mechanism [49]. This further amplifies inflammation, contributing to increased hsCRP levels since its synthesis is regulated by IL-6 and TNF-α [50]. Elevated levels of TNF-α and IL-6 may impair insulin signaling pathways, interfering with the anti-inflammatory effect of insulin, which in turn might promote inflammation [51].

There are several strengths and limitations of our study. This study was based on a large, well-defined clinical and homogeneous study cohort, and to our knowledge, it is the first study examining the association between early pregnancy hsCRP levels and the number of abnormal glucose values and type of hyperglycemia. Any autoimmune and/or infectious disease diagnoses and the GDM status of each participant were confirmed from the medical records, and potential confounders were considered in the analyses. As a limitation, even though an ethnically homogeneous study population is a strength of our study, the results may not be universally applicable to other ethnic groups.

## Conclusion

Serum hsCRP levels in early pregnancy were associated with subsequent GDM, suggesting that chronic inflammation preceding pregnancy may play a role in the development of GDM. The association was found especially with several abnormal values and postprandial hyperglycemia in the OGTT and was in part attributable to higher BMI among those who developed GDM. These findings highlight the role of chronic inflammation, and the clustering of metabolic risk factors involved in GDM and its severity.

## Electronic supplementary material

Below is the link to the electronic supplementary material.

Supplementary Material 1

## Data Availability

No datasets were generated or analysed during the current study.

## Abbreviations

BMI: Body mass index
CVD: Cardiovascular diseases
CI: Confidence interval
GDM: Gestational diabetes
hsCRP: High-sensitivity C-reactive protein
ICD-10: International Statistical Classification of Diseases and Related Health Problems: Tenth Revision
IGT: Impaired glucose tolerance
OGTT: Oral glucose tolerance test
OR: Odds ratio
SD: Standard deviation
T2D: Type 2 diabetes
