# Supplementary material for: Associations of early pregnancy high-sensitivity C-reactive protein levels with subsequent gestational diabetes: A Finnish gestational diabetes study
Source: Diabetol Metab Syndr. 2025 Jul 16;17:266. doi: 10.1186/s13098-025-01843-0 (PMC12269149; doi:10.1186/s13098-025-01843-0)
Supplement: Supplementary file 1 — Supplementary Material 1 [file 13098_2025_1843_MOESM1_ESM.docx]

**Associations of early pregnancy high-sensitivity C-reactive protein levels with subsequent gestational diabetes: A Finnish Gestational Diabetes study**

**Supplementary Table 1. Diagnostic classification of autoimmune diseases considered according to the International Classification of Diseases, 10th revision (ICD-10)**

| **Subgroups of autoimmune diseases** | **Diagnosis** | **ICD10 codes** |
| --- | --- | --- |
| Blood autoimmune diseases | Pernicious anemia | D51.0 |
|  | Autoimmune hemolytic anemia | D59.1 |
|  | Idiopathic thrombocytopenic purpura | D69.3 |
|  | Sarcoidosis | D86 |
| Endocrine autoimmune diseases | Thyrotoxicosis | E05 |
|  | Autoimmune thyroiditis | E06.3 |
|  | Primary adrenocortical insufficiency | E27.1 |
| Nervous system autoimmune diseases | Multiple sclerosis | G35 |
|  | Guillain-Barré syndrome | G61.0 |
|  | Myasthenia gravis | G70.0 |
| Gastrointestinal autoimmune diseases | Chronic atrophic gastritis | K29.4 |
|  | Crohn disease | K50 |
|  | Ulcerative colitis | K51 |
|  | Autoimmune hepatitis | K73 |
|  | Primary biliary cirrhosis | K74.3 |
|  | Primary sclerosing cholangitis | K83.01 |
|  | Celiac disease | K90.0 |
| Skin autoimmune diseases | Pemphigus | L10 |
|  | Pemphigoid | L12 |
|  | Psoriasis | L40 |
|  | Alopecia areata | L63 |
|  | Vitiligo | L80.9 |
|  | Pyoderma gangrenosum | L88 |
| Connective tissue autoimmune diseases | Rheumatoid arthritis | M05–M06 |
|  | Juvenile arthritis | M08 |
|  | Polyarteritis nodosa | M30.0 |
|  | Wegener granulomatosis | M31.3 |
|  | Giant cell arteritis with polymyalgia rheumatica | M31.5 |
|  | Other giant cell arteritis | M31.6 |
|  | Systemic lupus erythematosus | M32.1, M32.9 |
|  | Dermatopolymyositis | M33 |
|  | Scleroderma | M34 |
|  | Sjögren syndrome | M35.0 |
|  | Polymyalgia rheumatica | M35.3 |
|  | Ankylosing Spondylitis | M45 |

**Supplementary Table 2. Diagnostic classification of infectious diseases and asthma considered according to the the International Classification of Diseases, 10th revision (ICD-10)**

| **Subgroups of infections** | **Diagnosis** | **ICD-10 codes** |
| --- | --- | --- |
| Respiratory infections | Infections in the ear | H60.0–H60.9 |
|  | Nasopharyngitis | H65.0–H67.9 |
|  | Sinusitis | A36.1 |
|  | Pharyngitis | J00.0–J00.9 |
|  | Tonsillitis | J01.0–J01.9 |
|  | Laryngitis and tracheitis | J32.0–J32.9 |
|  | Acute upper respiratory infections of multiple and unspecified sites | J02.0–J02.9 |
|  | Peritonsillar abscess | A36.0 |
|  | Influenza | J03.0–J03.9 |
|  | Pneumonia | J35.0 |
|  | Other acute lower respiratory infections | A36.2 |
|  | Abscess of lung | J04.0–J05.9 |
| Gastrointestinal infections | Intestinal infectious diseases | A00.0–A09.9 |
|  | Hepatitis | A42.1 |
|  | Gastritis and duodenitis | B15.0–B19.9 |
|  | Appendicitis | K29.0–K29.9 |
| Genitourinary infections | Acute nephritic syndrome | N00 |
|  | Nephritis | N08.0 |
|  | Cystitis | N10.0–N10.9 |
|  | UTI, site not specified | N30.0 |
|  | Inflammatory diseases of female pelvic organs | N39.0 |
| Sexually transmitted diseases | Syphilis | A50.0–A53.9 |
|  | Gonorrhea | A54.0–A54.9 |
|  | Chlamydia | A55.0–A56.9 |
|  | Anogenital herpes | A60.0–A60.9 |
|  | HIV/AIDS | B20.0–B24.9 |
|  | Anogenital warts | A58.0–A58.9 |
|  | Other sexually transmitted infections | A63.0 |
| Skin and subcutaneous tissue infections | Erysipelas | A46.9 |
|  | Viral warts | B07.9 |
|  | Dermatophytosis and other superficial mycoses | B35.0–B36.9 |
|  | Cellulitis and abscess | L02.0–L03.9 |
|  | Acute lymphadenitis | L04.0–L04.9 |
|  | Pilonidal cyst | L05.0–L05.9 |
|  | Other local infections of skin and subcutaneous tissue | A36.3 |
| Infections during pregnancy | Infections of genitourinary tract | O23.0–O23.9 |
|  | Other infections arising during pregnancy | O98.1–O98.9 |
| Other infections | Certain bacterial diseases | A20.0– A29.9 |
|  | Spirochetal disease | A32.0–A35.9 |
|  | Rickettsiosis | A38.0–A41.9 |
|  | Viral infections | A42.2–A44.9 |
|  | Mycoses | A48.0 |
|  | Protozoal diseases, helminthiases, pediculosis, acariasis and other infestations | A48.2–A49.9 |
|  | Unspecified infectious diseases | A65.0–A69.9 |
|  | Infections of endocrine organs | A75.0–A79.9 |
|  | Infections of nervous system | A80.0–A99.9 |
|  | Infections of circulatory system | B00.0–B02.9 |
|  | Other infections of gastrointestinal system | B05.0–B06.9 |
|  | Infections of musculoskeletal system and connective tissue | B08.1–B09.9 |
| Asthma J45 | | |

**Supplementary Figure 1. The directed acyclic graph summarizing the hypothetical causality between GDM and hsCRP, and potential confounding variables used in the regression analyses.**

**
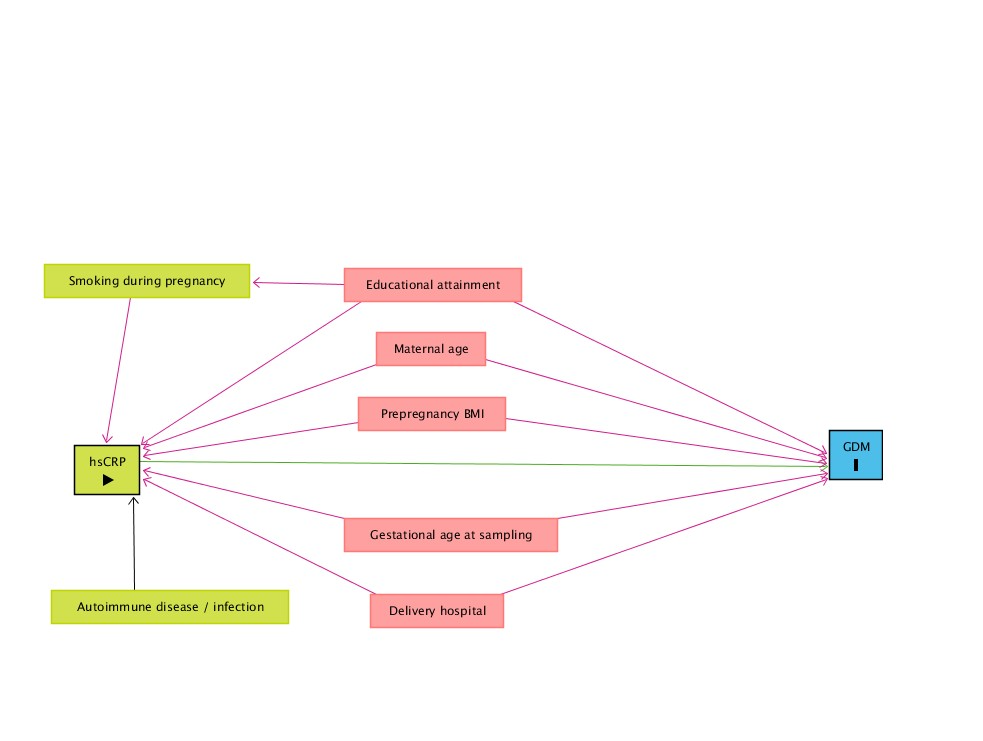
**

The green (►) square (hsCRP) represents exposure, the blue (I) square (GDM) is the outcome, the pink rectangles are the precursors of exposure and the outcome (confounders) [gestational age at sampling and delivery hospital are “technical” confounders; educational attainment, maternal age, prepregnancy BMI are other confounders], the green arrow demonstrates the hypothetical causal path, and the pink arrows demonstrate biasing paths.
